# 3D biofabrication of diseased human skin models in vitro

**DOI:** 10.1186/s40824-023-00415-5

**Published:** 2023-08-22

**Authors:** Minjun Ahn, Won-Woo Cho, Wonbin Park, Jae-Seong Lee, Min-Ju Choi, Qiqi Gao, Ge Gao, Dong-Woo Cho, Byoung Soo Kim

**Affiliations:** 1 Medical Research Institute, Pusan National University, Yangsan, 626841 Kyungnam Korea; 2 Department of Mechanical Engineering, Pohang University of Science and Technology, Pohang, Republic of Korea; 3 School of Biomedical Convergence Engineering, Pusan National University, Yangsan, Republic of Korea; 4 School of Medical Engineering, Beijing Institute of Technology, Beijing, 100081 China

**Keywords:** Skin engineering, Diseased-skin model, Tissue engineering, In vitro modeling

## Abstract

Human skin is an organ located in the outermost part of the body; thus, it frequently exhibits visible signs of physiological health. Ethical concerns and genetic differences in conventional animal studies have increased the need for alternative in vitro platforms that mimic the structural and functional hallmarks of natural skin. Despite significant advances in in vitro skin modeling over the past few decades, different reproducible biofabrication strategies are required to reproduce the pathological features of diseased human skin compared to those used for healthy-skin models. To explain human skin modeling with pathological hallmarks, we first summarize the structural and functional characteristics of healthy human skin. We then provide an extensive overview of how to recreate diseased human skin models in vitro*,* including models for wounded, diabetic, skin-cancer, atopic, and other pathological skin types. We conclude with an outlook on diseased-skin modeling and its technical perspective for the further development of skin engineering.

## Background

**Fig. 1:**
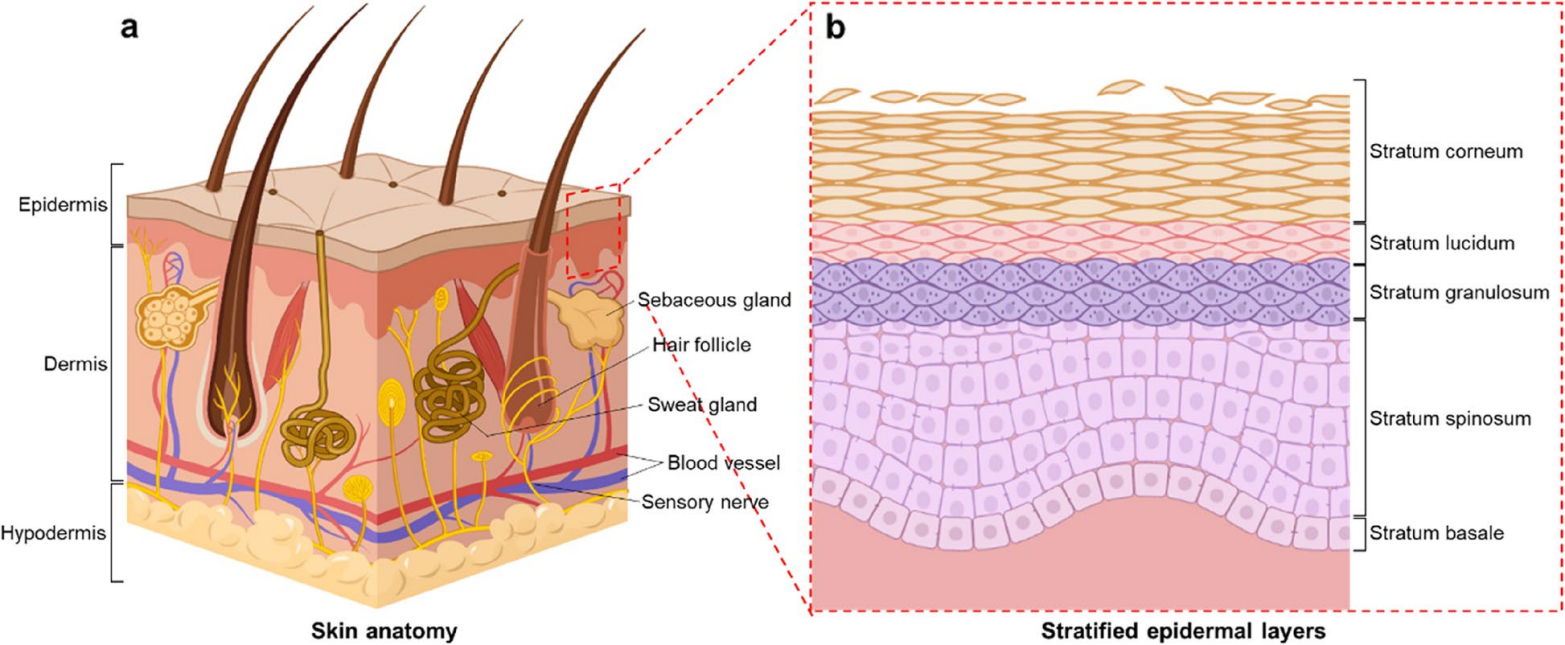
Schematic representing the skin structure. **a** Anatomy of human skin. **b** Stratified epidermis consisting of five distinct layers

Human skin is a complex organ in which various types of cells intimately interact with each other. It primarily consists of three layers: the hypodermis, dermis, and epidermis [1–3] (Fig. 1a). Each layer has a unique structure and cell/matrix composition. The hypodermis, known as the subcutaneous adipose tissue, is located in the innermost layer and connects the skin to the muscle or bone. It is a highly vascularized tissue and is densely packed with adipocytes that are responsible for energy homeostasis [4, 5]. The dermis is located between the hypodermis and epidermis. Its main cell type is fibroblasts, which produce collagen fibers and elastin that are aligned parallel to the skin surface [2, 6]. These matrix components provide excellent elastic properties for the skin [7]. In addition, various appendages, including blood vessels, hair follicles, sensory nerves, sweat glands, and sebaceous glands, are present in the dermal layer. The epidermis is the outermost layer of the human body. It predominantly comprises epidermal keratinocytes. Keratinocytes proliferate and begin to differentiate from the basal layer, and they form five stratified sublayers: the stratum basale, stratum spinosum, stratum granulosum, stratum lucidum, and stratum corneum (Fig. 1b) [8]. During the epidermal differentiation process, keratinocytes considerably change their morphology and produce several biological factors including keratin, cytokines, and growth factors [9].

The skin not only functions as a barrier against physical stimuli from exterior environments but also protects the body from the encroachment of microbes or harmful invaders. The skin comprises an essential immune system; thus, if pathogens invade injured skin, the skin secretes antimicrobial proteins, such as defensins, to prevent the growth and colonization of bacteria [10, 11]. The skin also plays a key role in endocrine functions that are systemically involved in physiological and pathological mechanisms throughout the body [12, 13]. In addition, the skin is responsible for thermal insulation, water balance, sensory reception, and reduction of the harmful effects of ultraviolet radiation [3].

Because these various physiological functions of the skin are directly linked to the overall health of the human body, extensive studies have been conducted on skin. More recently, with the gradual increase in interest in skin health, bioengineered-skin models with pathological hallmarks have been developed as dermatological and pharmacological platforms to discover underlying mechanisms and evaluate new drugs. Animal models have traditionally been used as testing tools owing to their structural resemblance to human skin; however, these models are not ideal. The inaccuracy of animal-model predictions has been consistently identified. These inaccuracies result from fundamental genetic differences between humans and animals and insufficient translation between animal models and actual pathophysiological systems in humans [14, 15]. Moreover, ethical problems are recognized as inevitable limitations in the use of animal models; therefore, the importance of reliable in vitro diseased-skin models is increasingly emphasized [16, 17]. Accordingly, in vitro diseased-skin models are urgently required to identify novel drugs and treatments in the fields of pharmacological and biomedical research. Therefore, many pioneering studies have actively investigated the development of in vitro diseased human skin models, including models for wounded skin, diabetic skin, skin cancer, and atopic skin. Moreover, different biofabrication strategies may be necessary for each skin type to represent its pathophysiological hallmarks in vitro. This review briefly summarizes the biofabrication of diseased-skin models and discusses the overall outlook of this research field.

## Biofabrication of in vitro human skin model

Prior to the successful development of diseased-skin models, biofabricated healthy human skin models were developed using various methodologies. These healthy skin models were developed to represent the anatomical and physiological traits of native skin for an efficient representation of skin functions. The following subsections introduce representative human skin models that have intrinsic characteristics of human skin.

### Reconstructed human epidermis models and full-thickness human skin model

**Fig. 2:**
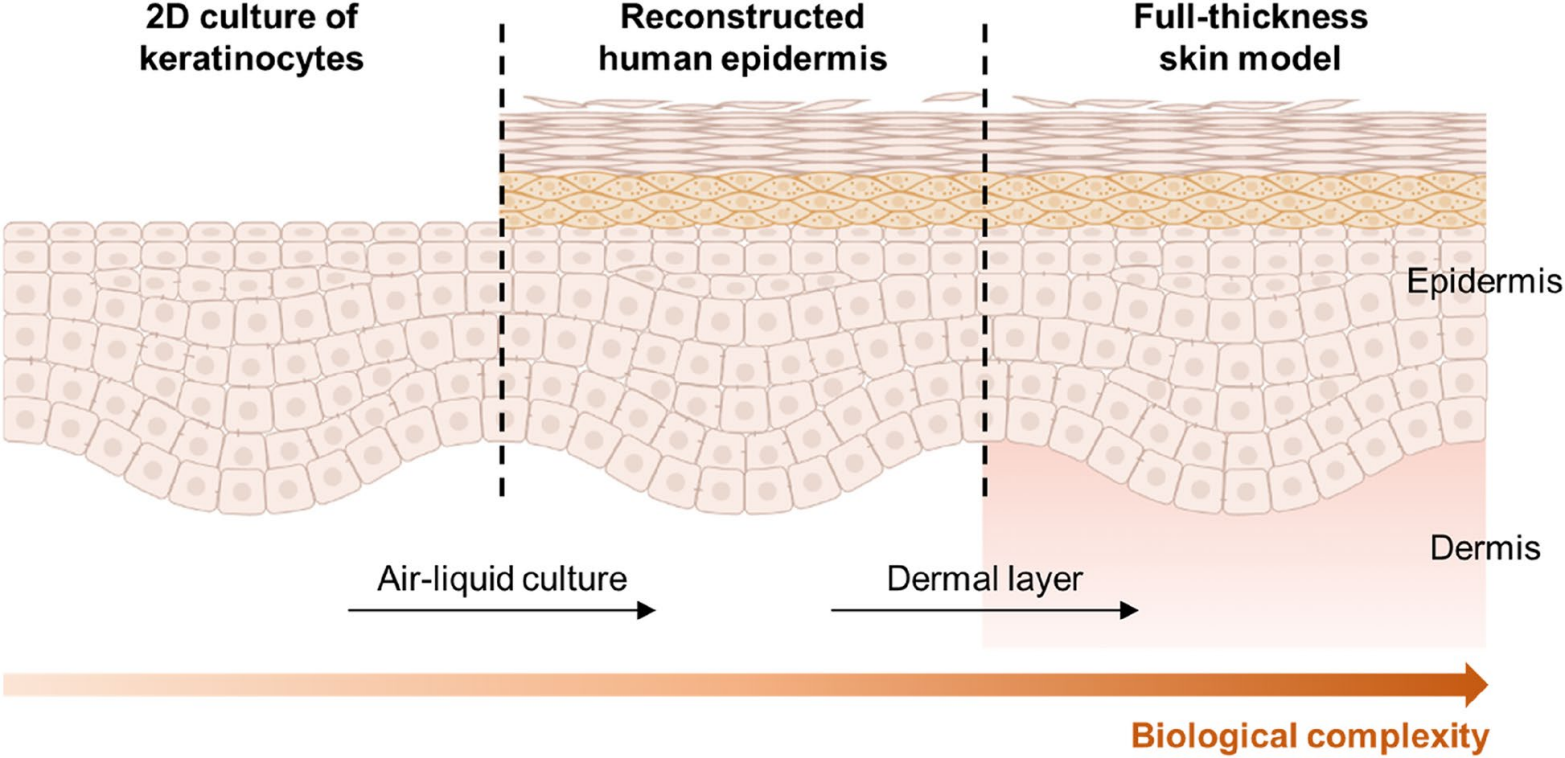
Advancement of an in vitro human skin model based on the tissue-engineering approach

Reconstructed human epidermis (RHE) models are in vitro human epidermis models consisting of a polycarbonate membrane, in which epidermal keratinocytes are cultured at an air–liquid interface, and through successful differentiation and cornification, they form epidermal sublayers (Fig. 2). In the 1990s, the first RHE models, EpiSkin® (L’ Oreal, Lyon, France) and EpiDerm® (MatTek Corp., Ashland, MA, USA), were developed and validated as testing models for skin corrosion by the European Centre for the Validation of Alternative Methods. After the Organisation for Economic Co-operation and Development (OECD) test guidelines were published in 2004, these models were evaluated for the prediction of skin irritancy [18]. Recently, various RHE models have been developed, such as Skinethic, EST1000, LabCyte Epi-model, OS-Rep, and StratiCell. To ensure independent reproducibility, the developed RHE models must be validated using various test systems, and they must fulfill the defined functional conditions. Related criteria are available from the OECD test guidelines (OECD TG 439). Because a majority of the barrier function of the skin is attributed by the stratum corneum, studies have focused on testing the barrier function of skin and the permeation patterns of various compounds and drugs using RHE models. do Nascimento Pedrosa et al. developed an RHE model for in vitro skin-irritation assays that followed OECD TG 439 [19]. Using this model, researchers tested the integrity and barrier function of the epidermis compared with those of native human epidermis. Moreover, they applied irritant and non-irritant materials to the RHE model to demonstrate its applicability in skin-irritation testing.

The majority of in vitro skin models for pharmaceutical purposes are based on RHE models; however, the dermal layer is another critical, yet neglected, feature in engineered-skin models. Fibroblasts, the major cellular component of the dermal layer, are responsible for forming connective tissue by producing an extracellular matrix (ECM), including collagens, fibrin, and fibronectin [20]. The standard cell-culture method showed that growth factors produced by fibroblasts promote keratinocyte growth [21]. Therefore, the interaction between fibroblasts and keratinocytes is essential for tissue morphogenesis and maintenance of the skin structure. This phenomenon was also observed in in vitro skin models, wherein fibroblasts play a crucial role in natural epidermal histogenesis and keratinocyte differentiation [22, 23]. Furthermore, fibroblasts are essential for wound healing and can increase keratinocyte resistance to toxic compounds. Therefore, developing a full-thickness (FT) in vitro skin model is essential to reflect physiological responses to toxic compounds.

The most common method for fabricating an in vitro FT skin model uses natural biomaterials such as collagen, chitosan, hyaluronic acid, gelatin, and fibrin. Casale et al. described a method in which dermal fibroblasts were cultured on gelatin-based scaffolds in a dynamic bioreactor to form a dermis layer composed of fibroblasts embedded in their own ECM [24]. Subsequently, keratinocytes were seeded on the surface of the dermal layer. Collagen is a major protein in the ECM and has been widely used in skin equivalents owing to its biocompatibility and vascularization potential. Reuter et al*.* developed an FT skin model using a collagen type-1-based hydrogel as a scaffold for culturing keratinocytes and fibroblasts [25]. However, the broad application of collagen is typically restricted, owing to its low mechanical strength. This results in significant contraction and a short lifespan resulting from fast degradation during in vitro culture [26–28]. Although the integration of keratin, fibrin, and gelatin provide enhanced mechanical properties and cell growth, these hydrogels cannot fully mimic the complex microenvironment of the skin. From this perspective, skin-derived decellularized ECM (skin-dECM) bioink has recently attracted increasing attention as an appealing source that offers physiologically relevant microenvironments. Kim et al*.* developed a porcine skin-dECM bioink and demonstrated its capability via in vitro and in vivo evaluations [15, 28]. The formulated bioink was used to construct an FT skin model, which exhibited a well-stratified epidermis and dermis after tissue maturation. However, various ECM components, such as proteoglycans, glycosamino glycans, fibronectin, collagens, and growth factors, may enhance its mechanical properties and cell functionality. Recently, proteomic analyses conducted by Han et al. revealed the specific matrisome protein composition in skin-dECM bioink, which plays a significant role in tissue-specific cellular behavior [29]. This tissue-specific microenvironment in skin-dECM bioink exhibited improved epidermal organization, fibroblast-derived ECM secretion, and barrier function compared with those of a collagen-based FT skin model. These results show that a developed skin-dECM bioink can be a promising source for engineered diseased-skin tissue and may offer better predictions for cosmetics and drug testing.

### 3D bioprinted skin model

Three-dimensional (3D) printing is a representative additive technology used to manufacture volumetric objects by simply depositing relevant materials layer by layer. Many engineers are attracted to its typically high speed and low fixed setup costs as well as its ability to create more complex geometries than those of traditional technologies with an ever-expanding list of materials [30, 31]. Based on this printing technique, 3D biological constructs can also be created using cell-laden biomaterials, which are frequently referred to as bioinks. This approach is generally termed 3D bioprinting and is classified by bioink-distribution types, including extrusion-based, laser-assisted, and droplet-based bioprinting. With advancements in printable biomaterials, 3D bioprinting has gradually gained popularity in in vitro skin engineering for numerous biological applications owing to its numerous advantages over conventional fabrication methods, such as the ability to reproduce complex structures with living cells, excellent repeatability, low costs, and high efficiency [32, 33].

Pourchet et al. demonstrated the bioprinting of complex 3D objects with fibroblasts [34]. They created FT skin (dermis and epidermis) by seeding human epidermal keratinocytes onto a bioprinted dermis. Lee et al. demonstrated that the 3D bioprinted skin model maintained its overall structure throughout the culture period, unlike the conventional manually fabricated skin model [35]. This may be because the layer-by-layer process offers a uniform cell distribution in the dermal compartment, thereby allowing the collagen fibers to be evenly dispersed by the contractile forces of fibroblasts.

**Fig. 3:**
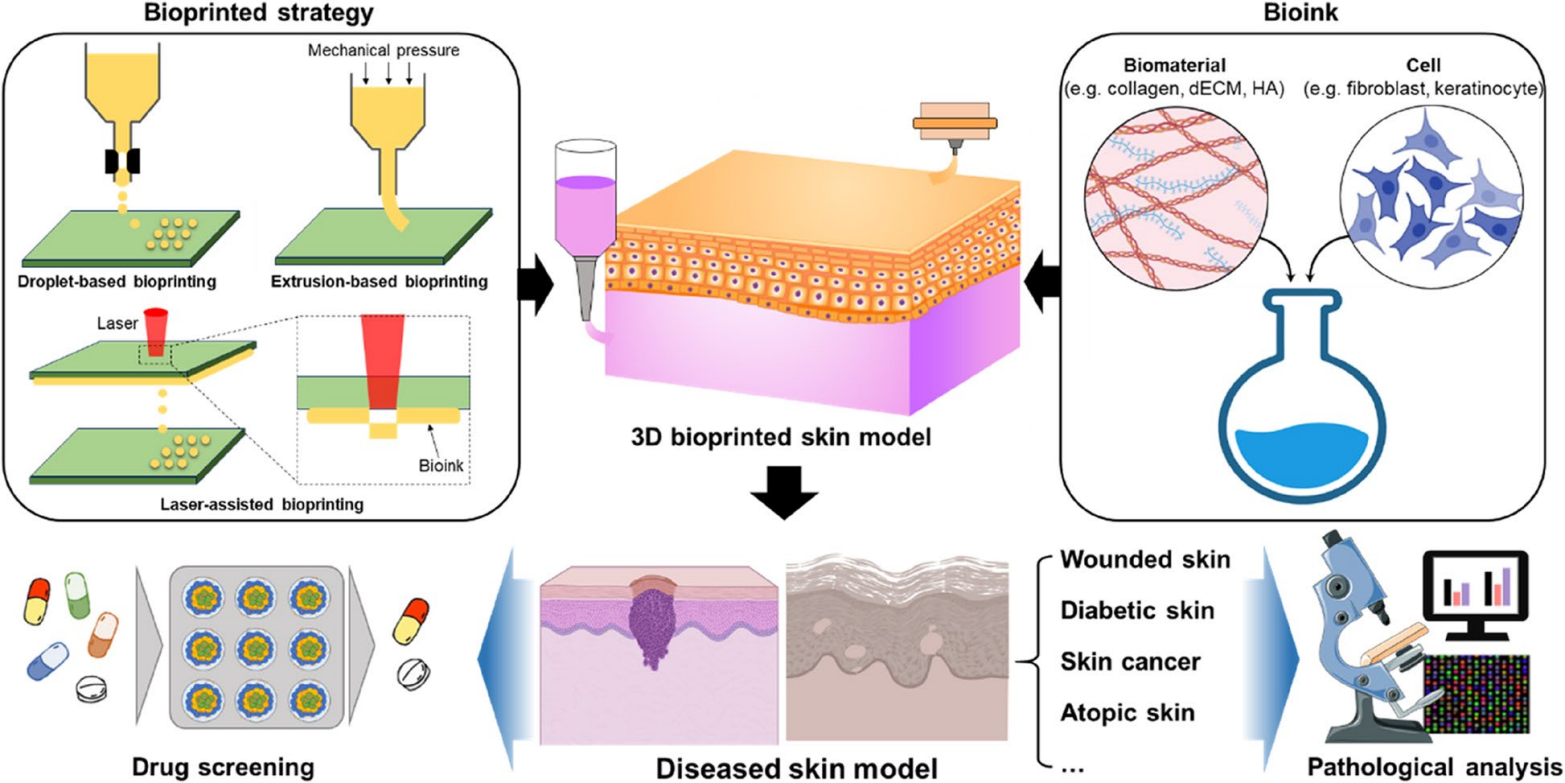
Schematic of the fundamental elements used for creating a 3D bioprinted skin model. These approaches can also be used for the biofabrication of diseased skin in vitro*,* which may be applied to drug screening and pathological analysis

Moreover, 3D bioprinting technology enables the creation of a heterogeneous skin model comprising the dermis and epidermis using multiple printing heads installed with various cell-laden bioinks. This method spatially controls the deposition of bioinks containing not only cells, but also the relevant proteins, growth factors, and other bioactive molecules to produce physiologically biomimetic skin models. Kim et al. presented a single-step-based bioprinting strategy to engineer a human skin model [36]. They installed extrusion and inkjet modules in a custom-made 3D bioprinter. The extrusion modules produced a fibroblast-populated dermis on a functional transwell system. Subsequently keratinocytes were evenly distributed on it using the inkjet module. This simple biofabrication method created mature skin models with a stratified epidermis that was similar to native human epidermis in terms of thickness and morphology. Beyond the formation of FT skin constituting only the dermis and epidermis, many studies have investigated the anatomical mimicking of bioprinted skin models by adding a hypodermis and appendages. Kim et al. increased the structural complexity of their skin model using 3D bioprinting techniques [15]. They incorporated a hypodermis comprising adipose tissue and perfusable vascular channels under the dermal compartment in their skin model. A perfusable vascular channel was created using the coaxial printing technique, thereby enabling the fabrication of tubular structures using a specialized printing nozzle. The nozzle resulted in the concentric deposition of multiple bioinks, and the bioink extruded at the core part consisted of sacrificial materials, such as Pluronic F127. Min et al. considered the actual anatomical structure of skin and printed a skin model that exhibited pigmentation by locating melanocytes between the dermis and epidermis layers [37]. Even though this method did not include treatment with ultraviolet irradiation or chemical stimuli, the 3D bioprinted skin model exhibited a freckle-like morphology at the dermal–epidermal junction. The aforementioned biofabrication tools can be applied to engineer both healthy- and diseased-skin models (Fig. 3).

## Biofabrication of diseased-skin model

Current skin-tissue engineering is not restricted to dermatological evaluation through accurate structural and physiological recreation but is expanding in the areas of in vitro modeling for the treatment of skin-associated diseases. These diseased-skin models can be used in research to uncover unknown pathological mechanisms as well as for the next generation of drug-screening tools, i.e., for predicting clinical outcomes using patient-derived cells. This section discusses various biofabricated skin models of wounds, diabetic skin, cancer, atopic dermatitis (AD), and other skin-related diseases.

### Wounded-skin model

One of the most prevalent skin diseases is external injury to the skin, including the epidermis and/or dermis, owing to physical stimuli such as burns, radiation exposure, and blunt force. Normal skin promotes wound healing in the lesion via hemostasis, growth, re-epithelialization, and remodeling by skin-constituting cells; otherwise, the damaged barrier functions of the wounded skin can cause another secondary infection [38, 39]. Wounded-skin modeling can be used to evaluate wound-healing processes.

**Fig. 4:**
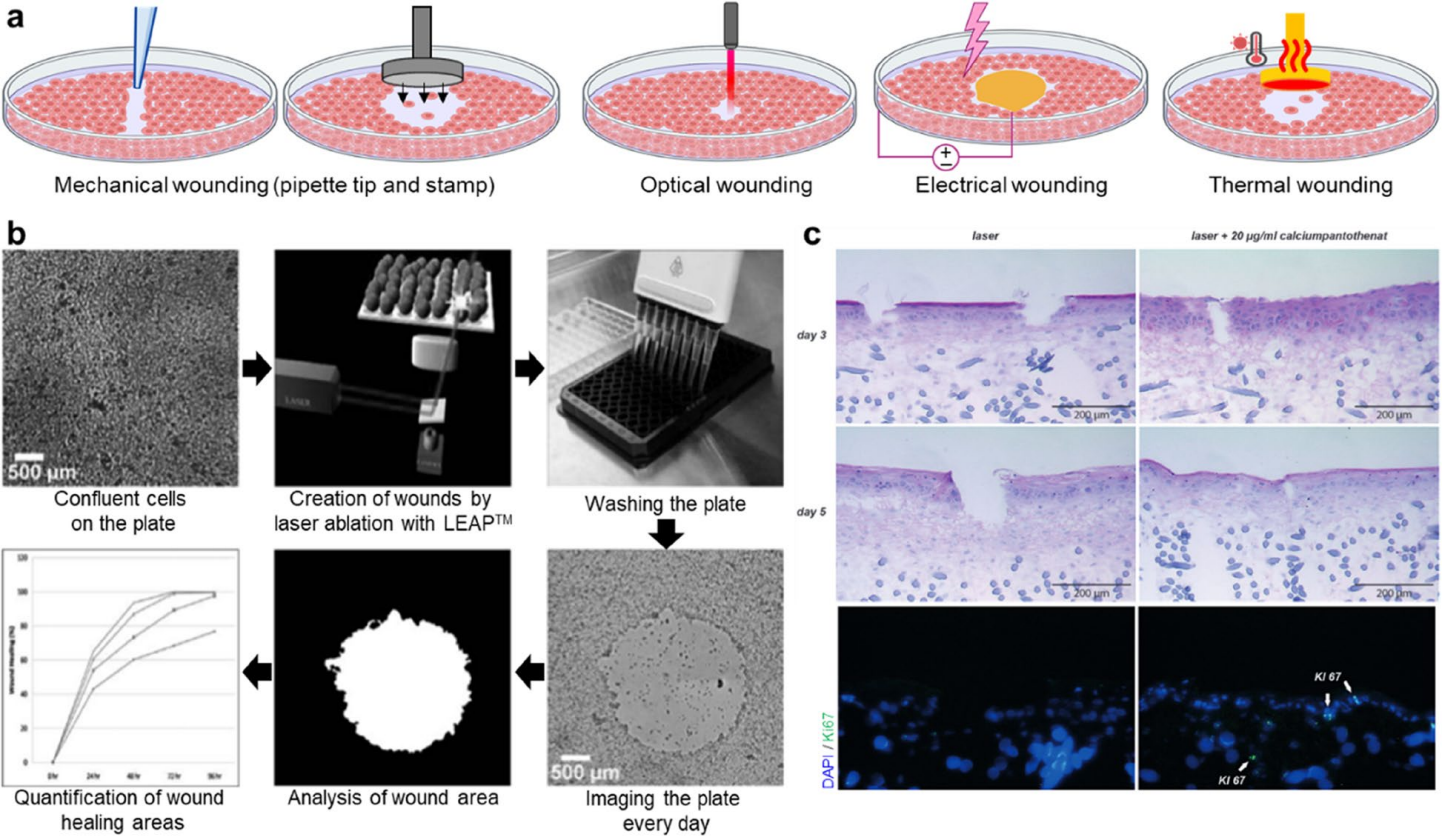
Representative studies on wounded-skin models. **a** 2D-based in vitro wound assays and wound-fabrication method using mechanical, optical, electrical, and thermal tools. **b** Workflow of wound-healing analysis using laser ablation [38]. First, cells were cultured on the plate until full confluency. A circular wound was then created using laser ablation with LEAP™, followed by rinsing to remove cell debris. Wound closure owing to cellular proliferation and migration was observed by LEAP™ in the bright-field mode daily. The wound area was calculated and quantified using the texture-segmentation algorithm developed by Smith et al. **c** Wound-healing analysis was conducted using a laser-irradiated skin model to demonstrate the effect of calcium pantothenate on re-epithelialization [42]. Reprinted with permission from Ref. [38, 42]

The most basic fabrication of wounded-skin models was achieved by culturing dermal fibroblasts and/or epidermal keratinocytes in two-dimensional (2D)-based culture plates. This method, termed in vitro wound scratch assay, created a cell-free region in a confluent cell monolayer using mechanical (pipette tip, cell scraper, metallic micro-indenter, and toothpick), optical (laser), electrical (electric cell-substrate impedance sensing), and thermal tools [40] (Fig. 4a). Mechanical wounding is typically used for cell ablation owing to its simple protocol. However, the scratches made by this method may be irregular because this method is conducted manually by a researcher. In addition, the surface coating may be removed, which facilitates the attachment of cells to the culture plates. Accumulation of the removed cells at the edge of the gap is another problem that may affect cell proliferation and migration, thereby producing inaccurate results. Alternatively, optical wounding is performed by irradiating a defined area of a confluent cell monolayer with a laser beam. A laser-enabled analysis and processing instrument (LEAP™, Cyntellect, San Diego, CA, USA) creates highly reproducible injuries under sterile conditions (Fig. 4b) [41]. Studies have monitored the healing process of filling artificially injured regions via cell migration using time-lapse microscopy.

Although 2D-based biofabrication approaches for wounded-skin models have provided a fundamental understanding of wound closure via cell migration and growth, 2D systems are limited in representing the structural similarity of the skin and actual 3D-based physiological responses in vivo. Moreover, cells cultured under 3D conditions exhibit appreciably different morphologies, cell–matrix/cell–cell interactions, and migration behaviors compared to those cultured under 2D conditions [40]. From this perspective, several efforts have been made to produce 3D wounded-skin models consisting of a bilayer structure, including dermal and epidermal compartments. For example, Safferling et al. standardized the fabrication process of a 3D wounded-skin model and its wound-healing assay [43]. After the maturation of the FT skin equivalent, wounding was caused at the center of the skin using a biopsy punch. A wounded skin compartment was then transferred to the top of an unpolymerized dermal compartment containing fibroblasts and incubated at 37 °C to bind the wounded skin and dermal compartment. However, this method is performed manually, which may increase the risk of contamination and result in poor wound reproductivity. To overcome these limitations, Rossi et al. developed an automated device that generated constant-sized wounds in every skin model in a sterile environment [44]. A wounded-skin model built using 3D bioprinting was also introduced. With the advantage of bioprinting, which produces a flexible design, a predesigned transwell system including a circular hole at the center was created for wounded-skin fabrication. After the formation of the FT skin on the transwell system, wounding was caused by automatically inserting a needle at a constant depth and speed into the hole. The tools used in mechanical wounding frequently cause the unintentional detachment of the epidermal layer. Marquardt et al. irradiated a CO_2_ laser on the FT skin model [42] (Fig. 4c). This non-sequential fractional laser inflicted identical damage to the epidermal layer in pre-defined regions. Their study showed that calcium pantothenate-containing media accelerated wound closure and upregulated Ki67 compared to the non-treatment results. Thus, wounded-skin models are typically used to analyze topically or systemically applied molecular compounds as a drug-testing platform. Currently, many studies are investigating various methodologies to form wounds on skin models to provide reliable outcomes, even if they do not create injuries in a living body.

### Diabetic-skin model

**Fig. 5:**
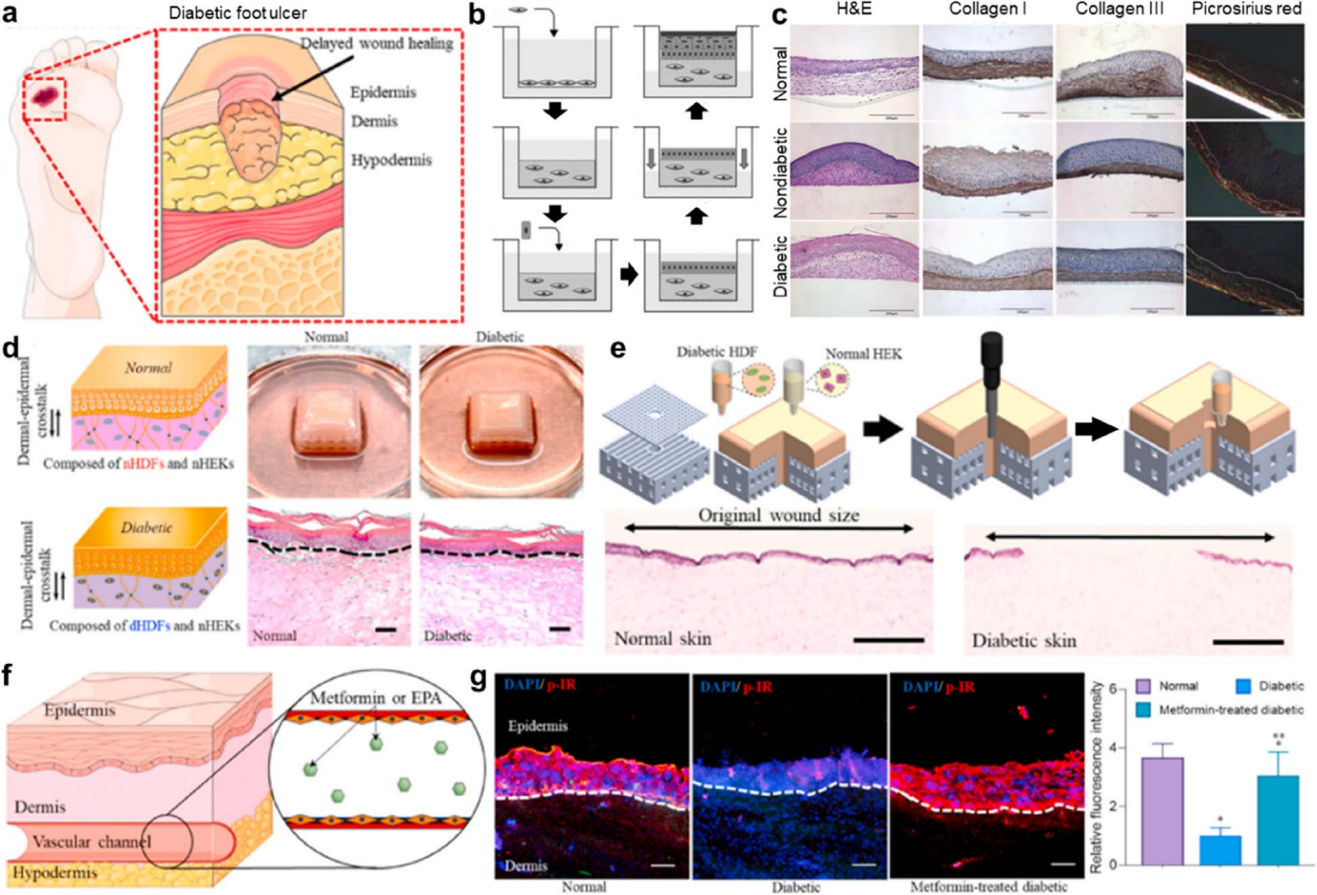
Representative studies for diabetic-skin models. **a** Schematic showing that even a small wound can become an ulcer owing to the poor wound-healing ability in patients with diabetes [47]. **b** Biofabrication process for the diabetic-skin model [48]. First, diabetic foot-ulcer fibroblasts contained in type I collagen were seeded onto the hanging cell-culture insert, which was followed by maturation for the dermal equivalent for three weeks of culture. Keratinocytes were then seeded on the dermal equivalent and allowed to proliferate for 5 d. Finally, an air–liquid interface culture was used to differentiate the keratinocytes, followed by the formation of stratified epidermal layers. **c** Comparison of skin models comprising human-foreskin, nondiabetic adult-foot, and diabetic foot-ulcer fibroblasts [48]. **d** Biofabrication strategy to create the diabetic-skin model via dermal–epidermal crosstalk [47]. **e** Wound fabrication using a 3D bioprinting device and wound-healing analysis via hematoxylin and eosin staining [47]. **f** Perfusion of metformin via a bioprinted vascular channel in the diabetic-skin model [47]. **g** Application of the diabetic-skin model as a drug-testing platform [47]. Reprinted with permission from Ref. [47, 48]

Diabetes mellitus, typically known as diabetes, is a metabolic disease that results in high blood-glucose levels. In a healthy body, the hormone insulin transfers glucose from the blood into cells to be stored or used for energy. However, a body with diabetes does not produce enough insulin or cannot effectively use insulin. The American Diabetes Association warns that skin disorders are the first noticeable feature of diabetes mellitus [45]. In general, patients with diabetes typically have several skin problems such as diabetic dermopathy, fungal infections, rash, blistering, and skin itching. These are results of poor blood circulation owing to high sugar levels in the bloodstream [46]. A diminished bloodstream impairs skin-cell functions, including cell proliferation, ECM remodeling, and self-healing (re-epithelialization). In particular, when a small wound is formed on the foot of a patient with diabetes, it readily develops into a diabetic foot ulcer, owing to poor wound-healing ability (Fig. 5a). This is a serious condition that requires amputation in 14%–24% of patients because effective treatments are lacking [46].

Many studies have investigated the development of in vitro models that represent a diabetic ulcer to examine its underlying pathogenesis and assess potential treatments for wound dressing. Maione et al. built a diabetic model based on a patient with diabetic foot-ulcer-derived fibroblasts [49]. Fibroblasts with diabetic features were encapsulated in a collagen type I matrix, and the model showed several hallmarks of chronic ulcers, including impaired angiogenesis, re-epithelialization, and ECM deposition. Smith et al. constructed three types of dermal matrices comprising human-foreskin, nondiabetic adult-foot, and diabetic foot-ulcer fibroblasts (Fig. 5b and c) [48, 50]. The dermal matrix was stabilized for over 7 d, and the results demonstrated that fibroblasts isolated from diabetic foot ulcers deposited an endogenous ECM de novo, which formed an environment similar to the one from which they were originally harvested. However, these studies did not compare the poor wound-healing capabilities, which constitute the major hallmark of diabetic ulcers, of the diabetic-skin model in vitro with those of a normal skin construct. Instead, they showed that monocytes co-cultured with diabetic skin constructs were differentiated into the pro-inflammatory M1 phenotype that is generally observed in patients with diabetic ulcers. A 3D-bioprinted diabetic-skin model was first developed in 2021 (Fig. 5d) [47]. This study established a bioengineering platform capable of constructing a skin model representing the pathological features of diabetes. The in-house built bioprinting platform could simultaneously locate biomaterials, including synthetic polymers and cell-laden bioinks, via extrusion- and inkjet-based modules. The diabetic dermal fibroblast-laden bioink was extruded on a customized transwell system, which was followed by an in vitro culture to form a relevant diabetic matrix. Keratinocytes harvested from a donor without diabetes were then uniformly distributed in the diabetic dermal compartment using an inkjet module. The study showed that a stratified mature epidermis was achieved via active interactions between diabetic fibroblasts and normal keratinocytes, and the impaired wound-healing ability was successfully represented in the diabetic-skin model when compared to that of the normal counterpart (Fig. 5e). Furthermore, the hypodermal compartment, which included a perfusable vascular channel, was incorporated beneath the skin model. This diabetic-skin model showed applicability for drug screening when the typical diabetic characteristics were alleviated via treatment with metformin, the most well-known drug for diabetes, through the vascular channel (Fig. 5f and g).

These studies confirmed that a skin model could accurately represent the skin environments of patients using patient-derived cellular components. This can further offer an effective methodology to recreate a diseased organotypic skin model as a promising testing platform based on ECM remodeling and cell–cell/cell–matrix interactions.

### Skin-cancer model

**Fig. 6:**
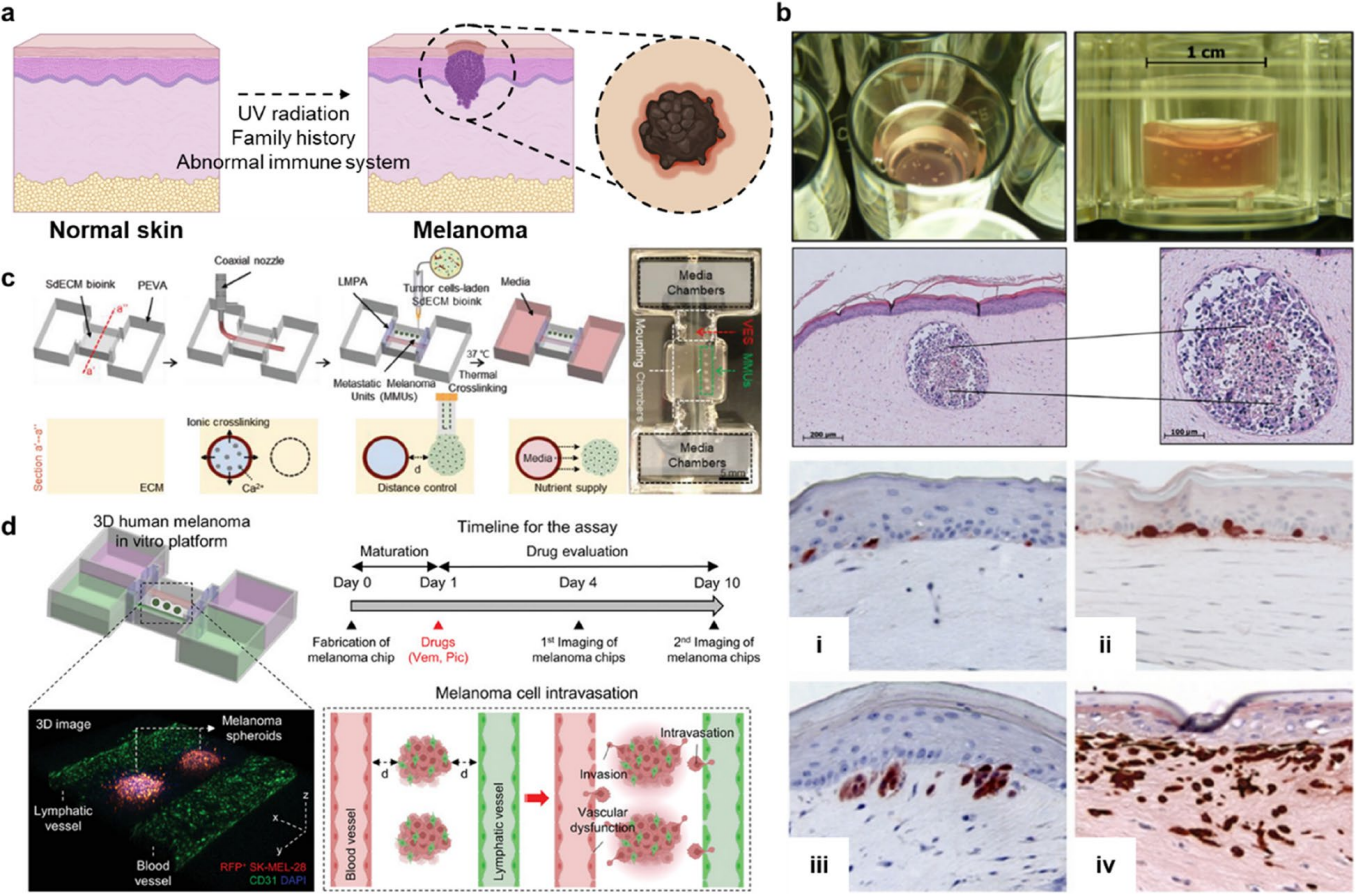
Examples of melanoma-skin models. **a** The main risk factors for melanoma. **b** Tissue-engineering approaches for the fabrication of in vitro melanoma models. Three-dimensional skin substitutes representing various stages of melanoma progression: (i) melanocyte located between epidermis and dermis; (ii) cell cluster formation by melanoma cells at the basement membrane; (iii) melanoma cell invasion into the dermis; (iv) aggressive invasion of melanoma cells into the dermis [53, 54]. **c)** In-bath bioprinting of melanoma cell aggregates with perfusable vascular channel [55]. **d)** In-bath bioprinting of melanoma stroma with blood and lymphatic vessel pair [56]. Reprinted with permission from Ref. [53–56]

The skin plays a vital role in protecting the internal organs from ultraviolet radiation; however, it is consequently exposed to ultraviolet radiation, which may result in skin cancer such as melanoma (Fig. 6a). Melanoma is one of the most aggressive and dangerous forms of skin cancer, and it has shown an increasing incidence over the last few decades. Existing melanoma models have been used to understand cancer pathology, perform drug testing, and bridge the gap between clinical trials and in vitro cancer models; however, developing in vitro melanoma models that reproduce the complex tumor microenvironment (TME) remains a challenge. The TME comprises various types of non-cancer cells and an extracellular matrix, which contribute to tumor progression in several ways [51, 52]. Therefore, tumors must be considered not just as a single unit but also as a heterogeneous aggregate in which numerous interactions occur. Moreover, tumor cells interact constantly with the surrounding cells and the acellular components of the tumor stroma that comprise the complex TME. Recently, research on the development of in vitro skin models that consider various TME factors has been actively conducted.

Müller et al. developed a 3D organotypic melanoma-skin model by integrating melanoma spheroids into a 3D human FT skin model (Fig. 6b) [53]. Using this model, Vörsmann et al. evaluated the sensitivity of melanoma cells to a tumor necrosis factor-related apoptosis-inducing ligand when ultraviolet B and cisplatin were applied simultaneously [57]. The developed melanoma model showed significant differences in therapeutic outcomes compared with those of the 2D condition. However, the size of the produced melanoma spheroids was nonuniform, and the labor-intensive fabrication process hindered the repeatability of the experimental results. In another study, Li et al. engineered a 3D melanoma-skin model to demonstrate the metastatic progression of melanoma in vitro [54]. Recently, Kim et al. devised an in-bath bioprinting technology for the uniform fabrication of metastatic melanoma spheroids (Fig. 6c) [55]. To print the metastatic melanoma spheroids, a high cellular density (> 10^8^ cells mL^−1^) of melanoma cells was encapsulated within a skin-dECM bioink. By incorporating the coaxial bioprinting technique, a perfusable vascular channel was fabricated together with spheroids to represent the cancer–vascular interaction in vitro. Recently, a lymphatic channel was added to the developed model to reproduce the complex TME with more accuracy while simulating the interaction between melanoma and the lymphatic system (Fig. 6d) [56].

### Atopic-skin model

**Fig. 7:**
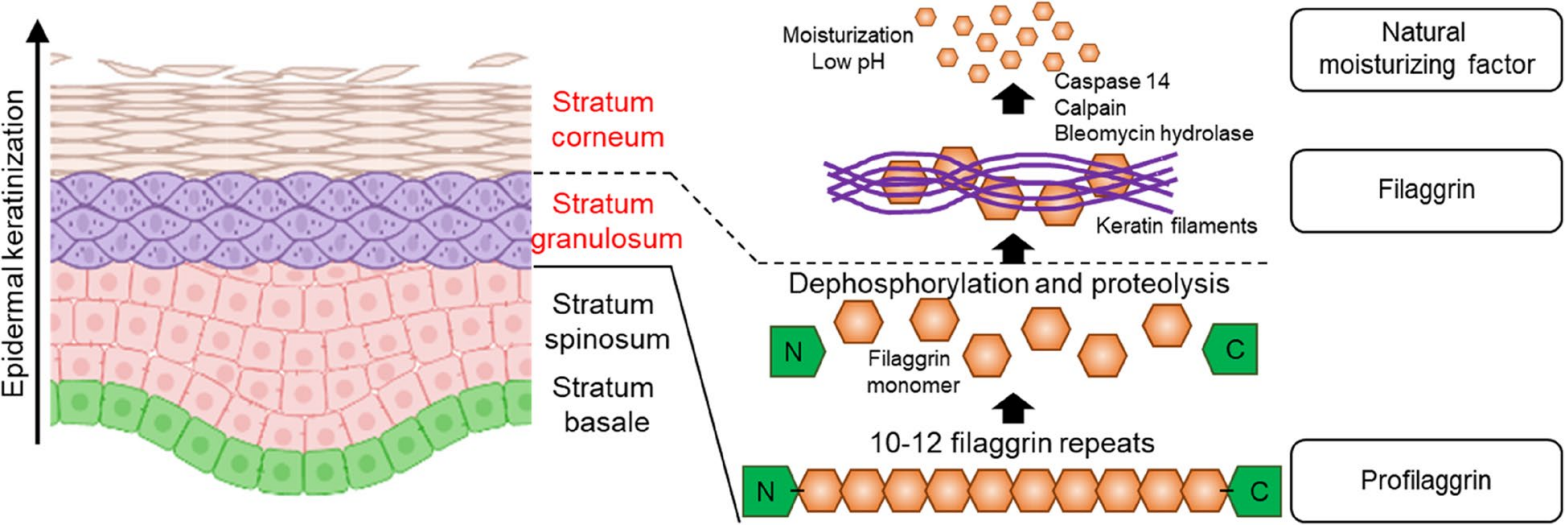
Pathology of AD. Filaggrin is located in the stratum granulosum of the epidermal layers. Profilaggrin is dephosphorylated and degrades into filaggrin monomers under the keratinocyte differentiation. Keratin filaments aggregate the cleaved filaggrin molecules, forming a dense matrix. The monomers are then degraded into natural moisturizing factors in the middle of the stratum corneum, which is regulated by proteases such as caspase 14, calpain, and bleomycin hydrolase

AD is the most common type of eczema, and it affects more than 9.6 million children and approximately 16.5 million adults in the United States. AD is a chronic inflammatory skin disorder that can persist for years or throughout life. Furthermore, it can overlap with other types of eczema. Intense pruritus (itchy skin) is known as the hallmark of AD, and it is generally accompanied by excessively dry erythematous lesions. Although epidermal-barrier alterations and T-helper 2 (Th2) immune-response dysregulation are recognized as the underlying triggers of AD pathogenesis, their complicated and multifactorial mechanisms make applying an adequate combination of treatments difficult (Fig. 7) [58, 59]. Therefore, developing biomimetic AD models for in-depth etiology as well as for screening new drugs/cosmetics is becoming increasingly important. Several AD-skin models have been developed in recent decades.

Conventionally, the 2D culture of a relevant cell type, such as immune cells and keratinocytes, has been considered the simplest tool for analyzing cellular biology associated with the pathogenesis of AD. Tatsuno et al. isolated peripheral lymphocytes from patients with AD to investigate gene expression in cell receptors and responses toward an epithelium-derived cytokine, thymic stromal lymphopoietin (TSLP), which is related to the Th2 immune reaction [60]. This 2D-based in vitro study demonstrated that TSLP receptors were partially expressed at the surface of the T cells harvested from patients with AD and that the interaction between the T cells and TSLP was strongly associated with the Th2 immune response. Furthermore, Jiao et al. co-cultured human dermal fibroblasts with effector immune cells, basophils and eosinophils, which are associated with human allergic inflammation, to demonstrate the mechanistic pathway in vivo in which pro-inflammatory cytokines/chemokines secreted from immune cells exacerbate AD [61]. The co-culture system showed a notable increase in vigorous inflammatory reactions to ligands related to Staphylococcus aureus, which reportedly cause the pathophysiology of AD. In particular, the study highlighted that direct cellular interactions between fibroblasts and basophils were required to elicit a meaningful response, whereas eosinophils could communicate with dermal fibroblasts through soluble mediators. In addition to immune cells, reduced expression of the filament-aggregating protein, filaggrin, was observed to initiate AD by culturing keratinocytes isolated from patients with AD [62]. Keratinocytes were differentiated using a culture medium containing representative Th2 cytokines that induced atopic responses, that is, interleukin (IL)-4 and IL-13. Filaggrin expression in an atopic-skin model was notably lower than that in normal skin. Based on the uncovered molecular mechanisms, the study concluded that atopic immune responses could be alleviated via the neutralization of IL-6 and IL-13, which eventually improved skin-barrier integrity. Moreover, transcriptional profiles of chemokine expression were investigated by comparing the outcomes of epidermal keratinocytes derived from AD and non-lesional skin [62, 63]. These studies have confirmed that the keratinocytes of patients with AD have inherently different chemokine profiles. For example, the keratinocytes of patients with AD exhibited upregulated levels of the granulocyte macrophage colony-stimulating factor, thereby contributing to the chronicity of AD lesions [63]. However, these simple 2D-based culture systems still cannot reflect the functional barrier or multiple interactions within actual AD skin; thus, further study requires the 3D modeling of reliable AD skin.

Most 3D AD-skin models that represent AD pathology at the epidermal level can be produced by culturing cytokine-based cocktails that are overexpressed in the epidermis. For example, Kamsteeg et al. stimulated the epidermis with Th2 cytokines, IL-4 and IL-13, and the resultant epidermal morphology exhibited spongiotic changes characterized by intercellular edema, which are typically observed in lesional AD patients [64]. These Th2 interleukin treatments resulted in apoptosis, spongiosis, and increased expression of AD epidermis-specific genes, such as carbonic anhydrase II and neuron-specific Nel-like protein 2 [65, 66]. Inflammatory molecules could also be added to the two Th2 interleukins to reduce filaggrin expression [67]. Vuyst et al. revealed that IL-25 in combination with Th-2 interleukins induced several hallmarks of the AD epidermis in the RHE model, including the augmentation of allergic inflammation [68]. Furthermore, cholesterol depletion from the membranes of keratinocytes could enhance morphological alterations because the removed lipid microdomains of the plasma membrane would guide the keratinocytes to become more sensitive to Th2 interleukins. In summary, IL-4 and IL-13 have been employed as pivotal cytokines to induce the hallmarks of the AD phenotype in the epidermis in vitro, and additional molecules can be considered according to the research of interest. In addition to treatment with interleukin-based cocktails, 3D organotypic AD-skin models have been established by hindering key gene expressions associated with epidermal-barrier formation. Notably, mutations in the filaggrin gene are known to be a predisposing cause of AD pathology; therefore, Kuchler et al. demonstrated that knocking down the filaggrin gene in keratinocytes constituting the skin model induced impaired epidermal differentiation, which resulted in spongiosis formation [69]. Moreover, they significantly increased the release of lactate dehydrogenase, IL-6, and IL-8 by applying sodium dodecyl sulfate to the filaggrin-knockdown skin model, which indicated the successful representation of skin irritation in vitro.

Although many pioneering studies have successfully represented several characteristics observed in patients with AD, a fundamental understanding of the etiological mechanisms of AD remains unclear. Therefore, while modeling diseased skin caused by complex and unknown pathologies such as AD, researchers must choose appropriate models based on their objectives from the multitude of studies performed with various rationales.

### Other pathological skin models

The bioprinting of in vitro skin models is necessary for engineering diseased-skin models as well as creating a platform for studying the pathology or efficacy testing of medications in the cosmetic industry. Vitiligo is a common skin disorder caused by the loss of melanin produced by epidermal melanocytes [70]. It is characterized by circumscribed white patches on the skin that tend to increase in size over time. The histological feature of vitiligo is the total absence of melanin and functioning melanocytes in the lesions, and inflammatory cells, CD4 + and CD8 + T lymphocytes, are typically observed on the edges of the lesions [71]. Therefore, reproducing the physiological features of vitiligo in vitro can provide valuable insights for understanding the mechanisms causing melanocyte loss and for testing potential treatment options for vitiligo patients. To ensure a homogeneous deposition of melanocytes in the dermis, Min et al. developed a 3D bioprinting technique capable of producing an FT skin model containing skin pigmentation [37]. After printing multiple layers of fibroblast-containing hydrogel, melanocytes and keratinocytes were sequentially printed over the dermis to induce skin pigmentation. The bioprinted skin model exhibited a well-stratified formation of the dermal and epidermal layers and the differentiation of keratinocytes into the stratum corneum. Moreover, melanocytes located at the dermal–epidermal junction exhibited freckle-like pigmentation in the absence of external stimuli. The developed printing technique also enabled the engineering of several types of pigmented-skin models, such as pigmentation and spot models. However, most studies related to vitiligo still focus on the histological approach, which is critically dependent on the selection of the biopsy site. To the best of our knowledge, a bioprinted skin model that represents the pathological characteristics of vitiligo has not yet been developed. Although the 3D vitiligo model represents the complex structure of the skin, it is rarely used owing to its high dependence on skilled expertise and experimental variations. However, 3D-bioprinting technology offers a reduction in experimental variables, thereby enabling the standardized fabrication of in vitro vitiligo models.

Similar to other diseases, vitiligo is caused by the interaction of various types of cells and ECM. Psoriasis is a complex chronic immune-mediated disease associated with the development of indurated inflammatory plaques on the skin [72, 73]. Studies proved that psoriasis is typically caused by the deregulated interaction between keratinocytes, the immune system, and the surrounding environment, which causes a consistent inflammatory response and activation of T cells. For a better understanding of the disease pathogenesis, elaborate psoriasis models that mimic the inflammatory environment of the disease are required so that they can be applied to develop therapeutics. Over the last few decades, in vitro FT skin models have been developed to mimic psoriatic skin. Saiag et al. examined the paracrine effect of psoriatic fibroblasts to induce the transformation of normal keratinocytes into a psoriasiform phenotype [74]. Owing to the secretion of soluble factors from psoriatic fibroblasts, enhanced keratinocyte outgrowth was successfully induced. Barker et al. [75] used a similar approach. Collagen-based skin substitutes were fabricated using psoriatic keratinocytes and fibroblasts and compared with substitutes made of normal cells. The data indicated increased cell proliferation and expression of pro-inflammatory cytokines tumor necrosis factor-α, interferon-γ, and IL-8 in psoriatic cells. Although psoriatic cells can induce the psoriasis phenotype in vitro, current research is limited by its reliance on self-assembly mechanisms for recreating psoriasis. Notably, using psoriatic cells and recreating a pro-inflammatory environment are both important for an enhanced representation of psoriasis. Moreover, to enhance reproducibility and reduce batch-to-batch variation of the model, 3D-bioprinting technologies can enable in situ development of pro-inflammatory conditions of the skin via the precise deposition of immune cells and growth factors.

## Conclusion

Over the past several decades, the development of skin models has demonstrated promising possibilities for replacing conventional animal studies that cause ethical problems as well as discrepancies in results owing to genetic differences. In conjunction with the complete prohibition of animal testing in the cosmetic industry since 2013, the importance of creating a reliable diseased-skin model has been reinforced in drug screening and pathological mechanism studies. This is challenging because diseased-skin engineering necessitates the embodiment of representative disorder characteristics based on a fundamental understanding of its pathology. However, advances in biofabrication methodologies, such as bioprinting, have enabled researchers to create structurally and functionally accurate diseased-skin models in vitro, thereby ensuring reliable outcomes. Furthermore, diseased-skin engineering has focused on the development of a cost-effective, high-throughput, and personalized platform with constant availability and scalability. Hence, biofabrication methods are becoming increasingly automated and specialized. This technical progress may aid in uncovering the underlying causes of skin diseases, which may further facilitate the discovery of suitable treatments.

## Data Availability

Not applicable.

## Abbreviations

RHE: Reconstructed human epidermis
OECD: Organisation for economic co-operation and development
ECM: Extracellular matrix
FT: Full-thickness
Skin-dECM: Skin-derived decellularized ECM
3D: Three-dimensional
AD: Atopic dermatitis
IL: Interleukin
2D: Two-dimensional
TME: Tumor microenvironment
Th2: T-helper 2
TSLP: Thymic stromal lymphopoietin
